# Supplementary material for: Antimicrobial-resistant pathogens on the plate: A semi-quantitative hygiene risk evaluation of raw beef consumption in Ethiopia within a One Health context
Source: PLoS One. 2026 Apr 7;21(4):e0346594. doi: 10.1371/journal.pone.0346594 (PMC13056190; doi:10.1371/journal.pone.0346594)
Supplement: Attachment — Submitted filename: Manuscript Number PONE-D-25-56353_review.pdf [file pone.0346594.s002.pdf]

The research is interesting and raises serious concerns about the potential public health risks associated with the consumption of raw beef in Ethiopia. I support its publication after appropriate modifications, as outlined below.

The names of bacterial species should be written in italics. There are some omissions, so please check carefully and correct them.

In microbiological food analysis, standardized methods require the use of control strains to ensure the validity of the results. Hence, it is necessary to specify which bacterial strains were used as controls (positive control and negative control, respectively).

Most antibiotics tested to assess the susceptibility of isolated strains, except ciprofloxacin, are not currently used to treat infections with *E. coli*, *Salmonella*, or *Shigella* due to recognized antimicrobial resistance, toxicity, and the availability of superior alternatives. Therefore, the authors should explain the criteria used to select the antimicrobials for this study.

The presence of *E. coli* strains in raw beef is not unusual, and not all strains of *E. coli* are potentially pathogenic (with the exception of *E. coli* O157:H7 or other STEC). In fact, *E. coli* is an indicator of hygiene in food industry, whose presence is allowed in a limited count, depending on the legislation or reference standard. Therefore, the authors should reconsider these statements in the discussion section.

In Conclusions section the authors need to insert a complex sentence highlighting the study limitation and further perspectives in the approached research area.
