# Supplementary material for: Antimicrobial-resistant pathogens on the plate: A semi-quantitative hygiene risk evaluation of raw beef consumption in Ethiopia within a One Health context
Source: PLoS One. 2026 Apr 7;21(4):e0346594. doi: 10.1371/journal.pone.0346594 (PMC13056190; doi:10.1371/journal.pone.0346594)
Supplement: Attachment — Submitted filename: Response to Reviewers.pdf [file pone.0346594.s004.pdf]

Response to reviewers and editorial comments

PONE-D-25-56353
Multidrug resistance on the plate: a health risk assessment of raw beef consumption in Ethiopia and the urgent need for integrated food safety policy in a One Health framework

We thank the editor and reviewers for their constructive feedback. Below, we provide point-by-point responses to each comment. Revisions were made accordingly and are indicated by exact line numbers based on the clean revised manuscript titled *Manuscript.*

1. Editorial comments

1. Please ensure that your manuscript meets PLOS ONE's style requirements, including those for file naming. The PLOS ONE style templates can be found at
   - ✓ https://journals.plos.org/plosone/s/file?id=wjVg/PLOSOne_formatting_sample_main_body.pdfan
   - ✓ https://journals.plos.org/plosone/s/file?id=ba62/PLOSOne_formatting_sample_title_authors_affiliations.pdf

   Response: *Revised according to PLOS ONE formatting requirements.*
2. Thank you for stating the following in the Acknowledgments Section of your manuscript: "Wollo University funded the data collection and laboratory investigation expenses of this research project, and the funder has no competing interest and has no role in publishing this paper." We note that you have provided funding information that is not currently declared in your Funding Statement. However, funding information should not appear in the Acknowledgments section or other areas of your manuscript. We will only publish funding information present in the Funding Statement section of the online submission form. Please remove any funding-related text from the manuscript and let us know how you would like to update your Funding Statement. Currently, your Funding Statement reads as follows: "Wollo University funded the data collection and laboratory investigation expenses of this research project." Please include your amended statements within your cover letter; we will change the online submission form on your behalf.
   Response: *Acknowledgment section removed and funding statement included in the appropriate section (Lines 420 to 426).*
3. We note that the grant information you provided in the 'Funding Information' and 'Financial Disclosure' sections do not match. When you submit, please ensure that you provide the correct grant numbers for the awards you received for your study in the 'Funding Information' section.
   Response: *Clarified and harmonized funding statement (Lines 420 to 426) and financial disclosure.*
4. Thank you for stating the following financial disclosure: "Wollo University funded the data collection and laboratory investigation expenses of this research project." Please state what role the funders took in the study. If the funders had no role, please state: "The funders had no role in study design, data collection and analysis, decision to publish, or preparation of the manuscript." If this statement is not correct you must amend it as needed. Please include this amended Role of Funder statement in your cover letter; we will change the online submission form on your behalf.
   Response: *Funders' role clarified in funding statement ((Lines 420 to 426).).*
5. Thank you for stating the following financial disclosure: "Wollo University funded the data collection and laboratory investigation expenses of this research project." We note that one or more of the authors is affiliated with the funding organization, indicating the funder may have had some role in the design, data collection, analysis or preparation of your manuscript for publication; in other words, the funder played an indirect role through the participation of the co-authors. If the funding organization did not play a role in the study design, data collection and analysis, decision to publish, or preparation of the manuscript and only provided financial support in the form of authors' salaries and/or research materials, please do the following:
   - ✓ Review your statements relating to the author's contributions and ensure you have specifically and accurately indicated the role(s) that these authors had in your study. These amendments should be made in the online form.
   - ✓ Confirm in your cover letter that you agree with the following statement, and we will change the online submission form on your behalf: "The funder provided support in the form of salaries for authors [insert relevant initials], but did not have any additional role in the study design, data collection and analysis, decision to publish, or preparation of the manuscript. The specific roles of these authors are articulated in the 'author contributions' section.

   Response: *Revised according to guidance; authors' roles and affiliations transparently declared.*
6. We note that your Data Availability Statement is currently as follows: "All data generated or analysed during this study are included in this published article and its supplementary information files." Please confirm at this time whether or not your submission contains all raw data required to replicate the results of your study. Authors must share the "minimal data set" for their submission. PLOS defines the minimal data set to consist of the data required to replicate all study findings reported in the article, as well as related metadata and methods (https://journals.plos.org/plosone/s/data-availability#loc-minimal-data-set-definition).
   For example, authors should submit the following data:
   - ✓ The values behind the means, standard deviations and other measures reported;
   - ✓ The values used to build graphs;
   - ✓ The points extracted from images for analysis.

   Authors do not need to submit their entire data set if only a portion of the data was used in the reported study. If your submission does not contain these data, please either upload them as Supporting Information files or deposit them to a stable, public repository and provide us with the relevant URLs, DOIs, or accession numbers. For a list of recommended repositories, please see https://journals.plos.org/plosone/s/recommended-repositories. If there are ethical or legal restrictions on sharing a de-identified data set, please explain them in detail (e.g., data containing potentially sensitive information, data are owned by a third-party organization, etc.) and who has imposed them (e.g., an ethics committee). Please also provide contact information for a data access committee, ethics committee, or other institutional body to which data requests may be sent. If data are owned by a third party, please indicate how others may request data access.
   Response: *Data availability statement updated; supporting files S1 and S2 added as per requirements (Lines 416-418, 433–443).*
7. If the reviewer comments include a recommendation to cite specific previously published works, please review and evaluate these publications to determine whether they are relevant and should be cited. There is no requirement to cite these works unless the editor has indicated otherwise.
   Response: *No literature suggested for citing.*

2. Reviewers' comments

Reviewer #1:

1) The research is interesting and raises serious concerns about the potential public health risks associated with the consumption of raw beef in Ethiopia. I support its publication after appropriate modifications, as outlined below.
Response: *Thank you.*
2) The names of bacterial species should be written in italics. There are some omissions, so please check carefully and correct them.
Response: *All species names have been revised and formatted in italics throughout the manuscript.*
3) In microbiological food analysis, standardized methods require the use of control strains to ensure the validity of the results. Hence, it is necessary to specify which bacterial strains were used as controls (positive control and negative control, respectively).
Response: *Control strains E. coli ATCC 25922 and S. aureus ATCC 25923 were included (Lines 183-184).*
4) Most antibiotics tested to assess the susceptibility of isolated strains, except ciprofloxacin, are not currently used to treat infections with E. coli, Salmonella, or Shigella due to recognized antimicrobial resistance, toxicity, and the availability of superior alternatives. Therefore, the authors should explain the criteria used to select the antimicrobials for this study.
Response: *Selection criteria for antibiotics clarified (Lines 173-178, 184-187, 396-398).*
5) The presence of E. coli strains in raw beef is not unusual, and not all strains of E. coli are potentially pathogenic (with the exception of E. coli O157:H7 or other STEC). In fact, E. coli is an indicator of hygiene in food industry, whose presence is allowed in a limited count, depending on the legislation or reference standard. Therefore, the authors should reconsider these statements in the discussion section.
Response: *Discussion revised to accurately reflect the hygienic indicator role of E. coli (Lines 344-346).*
6) In Conclusions section the authors need to insert a complex sentence highlighting the study limitation and further perspectives in the approached research area.
Response: *A limitation section was added (Lines 390-401).*

Reviewer #2:

1) This manuscript by Gebeyehu et al. addresses an important and timely public health issue: antimicrobial-resistant foodborne pathogens associated with raw beef consumption in Ethiopia, framed within a One Health perspective. The topic is relevant, and the dataset appears substantial. However, In its present form, the manuscript does not yet meet the standards for publication in a high-impact, international journal. I invite the authors to address the following concerns, specifically:
Response: *Thank you.*
2) The manuscript claims to conduct a "health risk assessment," yet the approach used is largely descriptive and semi-quantitative, without a clearly defined or validated risk assessment model. The risk scoring system (likelihood × severity) is insufficiently justified, not formally validated, and relies heavily on subjective scoring of hygiene practices. The manuscript does not clearly articulate what is novel relative to prior Ethiopian studies on raw meat contamination and AMR. Several similar prevalence studies are cited, but the incremental contribution of this work remains unclear. Therefore, the authors must clearly define whether this study is a microbiological prevalence study, an AMR surveillance study, or a formal risk assessment.
Response: *The manuscript now clearly defines the study as a semi-quantitative microbial risk estimation study (Lines 83-86, 190-205).*
3) If it is a risk assessment, the authors must justify the framework, explain assumptions, and clarify how it differs from existing Codex/WHO quantitative or semi-quantitative approaches. In addition, they must explicitly state the novel contribution in the Introduction and Discussion sections.
Response: *Risk scoring model and rationale were clarified in Methods section (Lines 218-226).*
4) Identification of Salmonella, Shigella, and E. coli O157:H7 relies entirely on culture and biochemical methods, without molecular confirmation. Given the high reported prevalence of Shigella and E. coli O157:H7, the absence of PCR or serological confirmation raises concerns about misclassification. Shigella is rarely isolated from meat compared to Salmonella and E. coli; the unusually high prevalence requires stronger validation. Therefore, the authors must acknowledge the limitations of culture-based identification explicitly and clarify whether any serotyping or molecular confirmation was attempted. Likewise, temper claims related to E. coli O157:H7 and Shigella prevalence or justify them with stronger methodological support.
Response: *Limitations of conventional identification and lack of molecular confirmation acknowledged (Lines 390-401).*
5) Within the antimicrobial susceptibility testing section, the antibiotic panel includes drugs of questionable relevance for Gram-negative enteric pathogens (e.g., erythromycin). Chloramphenicol is reported as 100% effective, yet this drug is largely obsolete or restricted in many settings due to toxicity. CLSI breakpoints for some antibiotics used (especially erythromycin) are not clearly applicable to the organisms tested. The authors must justify the choice of antibiotics based on national treatment guidelines or surveillance relevance and clarify CLSI breakpoint applicability for each organism–antibiotic combination. What about chloramphenicol results and their interpretation? Avoid presenting it as a practical treatment option.
Response: *Justification for antibiotic selection and interpretive thresholds included (Lines 173-178). I want to confirm that chloramphenicol is still used in the study area, especially for eye related infections.*
6) The hygiene scoring system (binary compliant/non-compliant) lacks validation and weighting. The reviewer wonder, why? The calculation of "cumulative gaps" (e.g., 52%, 76%, 100%) is arbitrary, with no sensitivity analysis. Risk categorization (low/medium/high) is asserted rather than statistically or probabilistically demonstrated. Please clarify these concerns, by providing a clear mathematical description of how risk scores were derived and justify thresholds for "high risk" using published frameworks.
Response: *Description of scoring method clarified, limitations noted in Discussion (Lines 83-86, 190-205, 390-401).*
7) Statistical methods are largely descriptive. Associations between hygiene practices and contamination are mentioned, but results of statistical tests are not presented (e.g., test statistics, p-values). Please clarify this issues!
Response: *Statistical analysis section revised, limitations due to subgroup size noted (Lines 212-217, 233-238, 390-401).*
8) The manuscript frequently uses strong causal language ("poses a serious health threat," "urgent need") without sufficient causal evidence.
Response: *Causal language has been tempered throughout.*
9) The One Health framing is conceptually invoked but not operationalized through data from animal health, environmental sampling, or policy analysis.
Response: *Discussion revised to reflect this limitation and suggest future research.*
10) Last, but not least, language and grammar require substantial professional editing.
Response: *The manuscript was carefully edited for clarity and language.*

Reviewer #3:

1) The manuscript discusses the public health risks of consuming raw beef in Ethiopia, especially in relation to bacterial contamination and antimicrobial resistance (AMR). The topic is highly relevant within the One Health concept, linking food safety, antimicrobial resistance, and consumer health. The manuscript provides useful primary microbiological data, hygiene observations, and risk assessment, making it potentially valuable for policymakers and public health researchers. Overall, this manuscript is well-written but needs some revisions and explanation. I recommend this manuscript for a major revision. I explain my concerns in more detail below. I ask that the authors specifically address each of my comments in their responses.
Response: *Thank you.*
2) Why were in this manuscript E. coli, Salmonella and Shigella prioritized? Please add deep explanation or references.
Response: *Pathogen selection rationale provided (Lines 69-80, 321-324).*
3) Research gap could be sharpened
Response: *Research gap clarified in the introduction (Lines 62-80).*
4) The introduction would benefit if the Authors can give clearer explanation about how impact the AMR to raw beef meat consumers.
Response: *Revised in introduction (Lines 62-86).*
5) Please add how to select 50 restaurant and 250 swab samples.
Response: *Sampling procedures clarified using municipal lists and stratified random sampling (Lines 100-114).*
6) Study design: The study is use cross-sectional, yet sampling occurred over a six-month period (January–June 2023). Clarification is needed on whether samples were collected once per site or repeatedly, and how temporal variability was addressed.
Response: *Timing and sampling strategy clarified (Lines 109-114, 116-120).*
7) 2.5.1 and 2.5.2 The authors should add molecular data like PCR, etc to confirm the bacteria and MDR.
Response: *Acknowledged as a limitation (Lines 390-401).*
8) Could the Authors explain, why for antibiotic resistance testing use 6 antibiotics? Please give the additional explanation.
Response: *Clarified in methods section (Lines 135-148).*
9) Why the Authors use Erythromycin and Amoxicillin? Because there are not relevant to the Gram-negative bacteria.
Response: *Justified based on local use and surveillance value (lines 395-397).*
10) Data analysis: Criteria for significance are not mentioned and the rationale for using Chi square & Fisher's test should be briefly stated.
Response: *Statistical analysis usage is revised from line 212-214, 233-243.*
11) Explanation in 3.3 more appropriate for the discussion
Response: *Text relocated where appropriate.*
12) The criteria used to define multidrug resistance (MDR) should be explicitly stated in the Results section before MDR percentages are reported.
Response: *MDR definition added in methods section (Lines 142-182).*
13) The Results section would be strengthened by presenting confidence intervals or measures of uncertainty for key prevalence estimates.
Response: *Because of the small sample size and low statistical power, inferential statistical analyses were not performed. Accordingly, the overall statistical analysis approach has been revised in the methods section.*
14) Although the Methods section mentions chi-square and Fisher's exact tests, the Results does not report corresponding test statistics, degrees of freedom, or p-values. Where associations are implied (e.g., between hygiene practices and contamination), statistical outcomes should be reported.
Response: *Addressed in revised statistical analysis (Lines 214-233) and limitations sections (390-401).*
15) Deeper analytical interpretation rather than descriptive comparison
Response: *Discussion revised accordingly.*
16) Insufficient acknowledgment of study limitations
Response: *Addressed in a dedicated limitations section (Lines 390-401).*
17) Please be careful with the statement of treatment failure or severe clinical outcomes because this study does not provide direct clinical evidence of infection, therapeutic failure, or adverse health outcomes. The data is only from meat and restaurant environments.
Response: *Language revised to reflect association, not causation.*
18) Please make it simply, the conclusion is too long. Conclude the result from your manuscript and add future research in this section.
Response: *Conclusion was condensed and reorganized (Lines 401-411).*
