# Supplementary material for: Antimicrobial-resistant pathogens on the plate: A semi-quantitative hygiene risk evaluation of raw beef consumption in Ethiopia within a One Health context
Source: PLoS One. 2026 Apr 7;21(4):e0346594. doi: 10.1371/journal.pone.0346594 (PMC13056190; doi:10.1371/journal.pone.0346594)
Supplement: Attachment — Submitted filename: Response_to_reviewers_auresp_2.pdf [file pone.0346594.s005.pdf]

PONE-D-25-56353R1

Antimicrobial-resistant pathogens on the plate: a semi-quantitative hygiene risk evaluation of raw beef consumption in Ethiopia within a One Health context

We sincerely thank the reviewer for the constructive feedback. We have carefully revised the manuscript to address all concerns. The key revisions and their locations are outlined below.

1. Reviewer 3 comments

1) The inclusion of erythromycin and amoxicillin in the antimicrobial susceptibility testing requires further clarification. Although the authors justify their selection based on local antimicrobial usage and surveillance value, previous studies consistently demonstrate that these antibiotics are generally ineffective against Gram-negative bacteria such as E. coli, Salmonella, and Shigella due to intrinsic resistance. Therefore, the scientific rationale for testing these agents should be more clearly articulated. If resistance is expected to approach 100% based on well-established intrinsic mechanisms, the purpose and added value of reporting these resistance rates need to be explicitly explained. The authors should also clearly state whether antimicrobial susceptibility testing was performed and interpreted according to internationally recognized standards, such as the Clinical and Laboratory Standards Institute (CLSI) or the European Committee on Antimicrobial Susceptibility Testing (EUCAST), and specify the breakpoints applied for each organism–antibiotic combination. If the inclusion of these antibiotics was intended to reflect local antimicrobial usage patterns (e.g., in veterinary practice), the manuscript should provide supporting data or appropriate references documenting such usage. Furthermore, it should be clarified whether intrinsically resistant agents were excluded from multidrug resistance (MDR) calculations to avoid potential overestimation of resistance levels. Without these clarifications, the interpretation, scientific validity, and epidemiological relevance of the susceptibility data remain uncertain.

***Response**: We acknowledge the reviewer's concern and have clarified the rationale for antibiotic selection. The manuscript now states that these antibiotics were included primarily for AMR surveillance within a One Health framework and to reflect local antimicrobial usage patterns, supported by WHO AGISAR guidance and relevant literature (lines 196–210). We also clarified that antimicrobial susceptibility testing followed CLSI standards and that breakpoint interpretations were applied only where validated; results for non-standard combinations are interpreted cautiously as surveillance data (lines 211–215). To avoid overestimation, intrinsic resistance was excluded from MDR classification (lines 216–219). Additionally, the Discussion now notes that erythromycin resistance may partly reflect intrinsic mechanisms (lines 433–436), and the limitations section clarifies that these antibiotics are not intended for direct therapeutic interpretation (lines 451–456).*

2) Discussion part

The Discussion section is currently largely descriptive and comparative in nature. Many of the interpretations follow the pattern of reporting prevalence values and comparing them with findings from other countries, followed by generalized explanations such as differences in hygiene practices. While such comparisons are useful, the section lacks sufficient analytical depth expected for publication in an international peer-reviewed journal.

The authors can give deep explanation about the results, for example:
- What biological or ecological factors could explain the unusually high prevalence?
- Could supply chain conditions contribute to the contamination?
- Does this suggest potential failure in cold chain management during transportation or storage?
- Whether the reported resistance (particularly to amoxicillin and erythromycin) reflects intrinsic resistance rather than acquired resistance.
- Whether there is evidence of antibiotic selection pressure in livestock production systems.
- The possible role of unregulated or excessive antimicrobial use in veterinary practice and many additional aspects that could be discussed based on your findings.

Response: *We have strengthened the Discussion by adding explanations of contamination pathways (lines 385–390), the role of cold chain and supply conditions (lines 391–394), and antimicrobial selection pressure in livestock systems (lines 417–422). We also improved interpretation by distinguishing intrinsic and acquired resistance (lines 433–437). These revisions enhance the analytical depth and align the manuscript with expectations for peer-reviewed publication.*
